# A dietary intervention with conjugated linoleic acid enhances microstructural white matter reorganization in experimental stroke

**DOI:** 10.3389/fneur.2024.1341958

**Published:** 2024-09-20

**Authors:** Frederike A. Straeten, Jan-Kolja Strecker, Anna-Lena Börsch, Bastian Maus, Maike Hoppen, Birgit Schmeddes, Lucia Härtel, Ann-Katrin Fleck, Stephanie van Zyl, Tabea Straeten, Carolin Beuker, Mailin Koecke, Louisa Mueller-Miny, Cornelius Faber, Gerd Meyer zu Hörste, Luisa Klotz, Jens Minnerup, Antje Schmidt-Pogoda

**Affiliations:** ^1^Department of Neurology, University Hospital Münster, University of Münster, Münster, Germany; ^2^Translational Research Imaging Center, University of Münster, Münster, Germany; ^3^Clinic of Radiology, University Hospital Münster, University of Münster, Münster, Germany

**Keywords:** conjugated linoleic acid (CLA), ischemic stroke, functional recovery, stroke neuroinflammation, MRI

## Abstract

**Background:**

A dietary supplementation with conjugated linoleic acid (CLA) was shown to attenuate inflammation and increase the proportions of circulating regulatory T cells (T_regs_) and M2-type macrophages in disease models such as autoimmune encephalitis and arteriosclerosis. Since T_regs_ and anti-inflammatory (M2-type) macrophages were found to enhance stroke recovery, we hypothesized that CLA-supplementation might improve stroke recovery via immune modulatory effects.

**Methods:**

Functional assessment was performed over 90 days after induction of experimental photothrombotic stroke in wild type mice (*n* = 37, sham *n* = 10). Subsequently, immunological characterization of different immunological compartments (*n* = 16), *ex vivo* magnetic resonance (MR, *n* = 12) imaging and immunohistochemical staining (*n* = 8) was performed. Additionally, we tested the effect of CLA *in vitro* on peripheral blood mononuclear cells from human stroke patients and healthy controls (*n* = 12).

**Results:**

MR diffusion tensor imaging (DTI) demonstrated enhanced microstructural reorganization of interhemispheric white matter tracts, dependent on lesion size. Functional recovery over 90 days remained unaffected. Detailed immunological analyses across various compartments revealed no significant long-term immunological alterations due to CLA. However, analyses of human blood samples post-stroke showed reduced levels of pro-inflammatory interferon-γ (IFN-γ) and tumor necrosis factor alpha (TNF-α) release by T-lymphocytes following *in vitro* treatment with CLA.

**Conclusion:**

We aimed to explore the efficacy of a dietary intervention with minimal known side effects that could be accessible to human stroke patients, regardless of the degree of disability, and without the risks associated with aggressive immunomodulatory therapies. Our main findings include improved microstructural reorganization in small infarcts and a reduced inflammatory response of human T cells *in vitro*.

## 1 Introduction

There is emerging evidence that not all impairments after stroke can be attributed to the primary ischemic lesion, but also to a secondary local and sustained inflammation, resulting in impaired connectivity and regional excitotoxicity due to deregulated neuronal coupling (1, 2).

While an initial immune response and recruitment of activated immune cells such as T_H_1-cells and neutrophils are needed at the acute stage of CNS injury, the sustained compartmentalized neuroinflammation hinders neuroregeneration and contributes to secondary deterioration (2, 3). This can largely be attributed to the secretion of pro-inflammatory cytokines by microglia and lymphocytes, including IFN-γ, TNF-α and IL-1β. T_regs_ counteract this detrimental cytokine elevation and contribute, mediated by IL-10, to the regulation of the deranged immune reaction (4).

Conjugated linoleic acid (CLA) encompasses a group of linoleic acid isomers with two conjugated double bonds, while immunomodulatory effects have been demonstrated predominantly for the cis-9,trans-11 (c9,t11) and trans-10,cis-12 (t10,c12) isoforms of CLA (5). CLA exerted an anti-inflammatory effect *in vivo* via activation of the peroxisome proliferator activator receptor (PPAR)-γ in immune cells, thereby skewing monocyte function to a more anti-inflammatory phenotype via induction of IL-10 *in vitro* and *in vivo*. This phenotype and its associated effects were beneficial in existent atherosclerosis (6). CLA has ameliorated the disease course of various experimental autoimmune diseases, especially of inflammatory bowel disease (7). Fleck et al. (8) showed beneficial effects of a CLA-rich diet in a mouse model of multiple sclerosis and attributed these effects to enhanced IL-10 production by murine myeloid cells and a suppressed T cell proliferation. For primary prevention of stroke, a 5% higher intake of linoleic acid accompanied by a lower intake of saturated fatty acids was associated with a reduced risk of ischemic stroke (9). Following stroke, the diversity of the gut microbiome is decreased and diversity of the gut microbiota is associated with an attenuated proinflammatory immune response (10). Omega-3 polyunsaturated fatty acids promoted white matter tract integrity in the corpus callosum as well as microglia-differentiation into anti-inflammatory (M2-)phenotypes and ameliorated long-term sensorimotor recovery in experimental stroke (11). The effect of CLA on stroke recovery has not been investigated so far.

To conclude, T_regs_ as well as a regulatory subset of myeloid cells are crucial for controlling the immune response after stroke and are thereby pivotal for stroke recovery. In myeloid cells, CLA has been shown to upregulate IL-10 production and to reduce secretion of IFN-γ (8). Supplementation of conjugated linoleic acid might be capable of suppressing the secretion of pro-inflammatory cytokines including TNF-α, IFN-γ, IL-17 and IL-6, and enhancing the secretion of anti-inflammatory cytokines, thereby polarizing myeloid and T cells into a more regulatory phenotype. This could bear the potential to counteract secondary local and sustained inflammation, which impairs stroke recovery and contributes to secondary neurodegeneration (2). In this exploratory study we aim to investigate a potential immune-modulating and neuro-regenerative effect of a CLA-rich diet in an experimental mouse model of ischemic stroke.

## 2 Methods

### 2.1 Experimental design and animals

The experiments conducted in this study complied with animal welfare regulations and were approved by the local governmental authorities, specifically the North Rhine-Westphalian Ministry for Environment, Health and Consumer Protection, Department for Animal Protection (81-02.04.2020.A326). The animals were provided by Charles River GmbH (Germany). Adult male mice from the wild type inbred mouse strain C57BL/6J, aged 12 weeks, were kept under standard laboratory conditions in the Central Animal Experimental Facility (ZTE), University Hospital Muenster. Animals were randomized into two groups receiving the following dietary interventions: The standard chow of the control group (*n* = 19) was C1000 (Altromin Spezialfutter GmbH & Co. KG) which interalia contained sunflower oil. For the treatment group (*n* = 18), the 11.1 g sunflower oil per kilogram of the C1000 diet was replaced by Tonalin^®^ TG80 (with CLA isomers: cis-9,trans-11 and trans-10,cis-12 at a share of 40% each, BASF/BTC-Europe GmbH, manufactured by Altromin Spezialfutter GmbH & Co. KG). The modified CLA-enriched chow (containing 0.8888% CLA) was fed from day of the photothrombotic stroke until the end of the experiment. Additional sham experiments were performed in which except for laser irradiation the exact same procedure as outlined in the following with injection of the same dose of Bengal Rose was performed. Each sham group consisted of 5 animals, sham procedure was performed according to Li et al. (12). The experimental design was fully randomized (lottery procedure).

### 2.2 Photothrombotic stroke

To induce photothrombotic stroke, the mice received Meloxicam (0.2 mg/kg bodyweight) as an analgesic 30 min prior to the procedure. Thereafter, mice were anesthetized using intraperitoneal ketamine hydrochloride (100 mg/kg body weight) and xylazine (10 mg/kg body weight) and kept at a constant body temperature of 37°C ± 0.5°C throughout the procedure. The skull was exposed following a dorsal scalp incision, and a laser beam was positioned 2 mm right of the bregma. The skull was irradiated by a continuous-wave laser (CoboltJiveTM75, Cobolt AB, Solna; Sweden) with a wavelength of 561 nm, an intensity of 30–34 mW, and an aperture of 4 mm for 20 min after intraperitoneal injection of 0.2 mL Rose Bengal (10 mg/mL).

### 2.3 Assessment of functional recovery

Neurological deficit scores were assessed using a previously described modified version of Menzies’ neuroscore (13), which ranges from 0 (no deficit) to 5 (death).

To assess sensorimotor deficits, the adhesive tape removal and the foot fault test were performed as previously described (14). The adhesive tape removal test was conducted as follows: For bilateral sensory stimulation, two adhesive dots (25 mm^2^) were placed on both palmar forepaws, and the time taken by the animals to remove the adhesive dots was recorded in three trials per day for each forepaw. The course over the experiment is shown by the latency in seconds to remove the adhesive dot from the paretic (left) paw and the healthy (right) paw. For analysis, the course of the latency of the left paw was respected.

To compare sensorimotor deficits, the foot fault test was employed. Animals were individually placed on an elevated 10 mm square wire mesh with a total grid area of 40 cm × 40 cm and were allowed to walk freely for 2 min while being videotaped. An investigator counted the number of foot faults and the total number of steps and calculated the ratio using the following formula: (number of foot faults/number of total steps) × 100. Both tests were performed at baseline, day 1 and then once per week.

The adhesive tape test was practised (once per day for three passes) during the acclimatisation period of 1 week before induction of the photothrombotic stroke. Thereby the animals got used to the laboratory facility and the test procedure and potential confounders due to stress was reduced. No animals were excluded at this stage.

### 2.4 MRI assessment

At 90 days post stroke, *ex vivo* diffusion-weighted magnetic resonance imaging (dMRI) allowed for whole-brain fiber tracking and tissue structure analysis through diffusion metrics. Brains in skulls were fixed in 4% para-formaldehyde (PFA) for 2–3 days, then transferred to 1% PFA doped with 2 mM Magnevist (Gd-DTPA, Bayer, Leverkusen, Germany) for additional 2–3 days with daily medium change. Whole skulls were embedded in 1% low-melting agarose (Sigma Aldrich, St. Louis, United States) doped with 2 mM Magnevist in 5 mL syringes. Samples were stored at 4°C before and after MRI. MR images were acquired at room temperature using a 9.4 T horizontal animal scanner with 20 cm bore (Bruker 94/20 USR BioSpec, Avance III, Bruker BioSpin, Ettlingen, Germany). The system is equipped with a 700 mT/m gradient system (BGA12S, BrukerBioSpin) and a 2-element cryogenic transceive coil (Bruker BioSpin) for image acquisition. The system is operated by ParaVision 6.0.1 (Bruker BioSpin). Images were acquired using a multishot 3D spin-echo EPI sequence with the following scan parameters: echo time = 24.5 ms, repetition time = 300 ms, 8 segments, bandwidth = 250 kHz, 100 × 100 × 100 μm^3^ resolution, gradient duration = 4.5 ms, gradient separation = 11 ms, 80 diffusion directions, *b*-values = 1,500 and 3,000 s/mm^2^.

Restricted diffusion was quantified using restricted diffusion imaging (15). Diffusion data were reconstructed using generalized q-sampling imaging (16) with a diffusion sampling length ratio of 1.4. A deterministic fiber tracking algorithm was used (17). Interhemispheric connectivity was assessed by manually placing a seed region at the midline corpus callosum (delineated by dominant left-right diffusion anisotropy, 50,000 seeds). The anisotropy threshold was 0.03, angular threshold 60°. The step size was 0.03 mm. Tracks with length shorter than 1 or longer than 20 mm were discarded. Lesion volumetry was performed on the dMRI scans from a manual outline of cortical voxels with signal voids or aberrant anisotropy vectors.

Fitting of diffusion metrics and fiber tracking were performed using DSI Studio.^1^

### 2.5 Tissue preparation

Ninety days after photothrombotic stroke, mice were perfused through the left ventricle with phosphate buffered saline for 5 min and 4% paraformaldehyde for 10 min under deep xylazine/ketamine anaesthesia. For immunohistochemistry, brains were removed, fixed in 4% paraformaldehyde overnight, immersed in 30% sucrose for 3 days, frozen and stored at −80°C.

### 2.6 Immunochemistry

Mounted coronal cryosections were incubated in blocking reagent (Roche Diagnostics) for 15 min to prevent unspecific protein binding. Subsequently, we used the following primary antibodies: anti-Laminin (Abcam, ab11575) with Alexa Fluor 549 donkey anti-rabbit (Life, A-21207) and Alexa Fluor 488 goat anti-mouse-IgG (heavy and light chain) (Life, A-11001). To reduce lipofuscin autofluorescence, we applied True Black^®^ Plus Lipofuscin (1:40 in DMSO, Biotium, 23014) for 10 min. Images were taken with a fluorescence microscope (Nikon Eclipse 80i, Nikon GmbH, Düsseldorf, Germany). For quantification of extravasated IgG, 8-bit fluorescence images were captured using a Nikon Eclipse 80i fluorescence microscope. Pixels between grey values 8–255 were considered positive and summed for each animal.

### 2.7 Flow cytometry

After induction of deep narcosis by overdosing anaesthesia, blood was taken from the right ventricle and transferred into a tube with 500 μL PBS (phosphate buffered saline)-heparine (4°C). The lymph nodes were taken up onto a plate with HBSS (Hank’s balanced salt solution) (4°C), the small intestine (without Peyer-patches) as well as the colon on a plate with HBSS (at room temperature). The brains were transferred into a 15 mL falcon filled with PBS (4°C).

Isolation of brain lymphocytes was performed in a sterile manner by means of Percoll^™^ density gradient centrifugation. Briefly, fresh collagenase (1:125) was added first to disintegrate the tissue/extracellular matrix. Subsequently, Percoll^™^ was added stepwise under repetitive centrifugation steps to obtain a continuous density gradient and the respective phase containing the brain lymphocytes were carefully pipetted off.

Cervical and mesenterial lymphocytes were isolated under sterile conditions by utilizing different cell strainer sizes to dissociate the lymphocytes from cells and tissue fragments of larger size. Cervical lymph nodes were transferred to a 70 μm plastic cell strainer and mesenteric lymph nodes were carefully homogenised by a metal strainer. Thereafter, strainers were washed with MACS buffer and after centrifugation, the pellet was resuspended in TexMACS^™^ medium (Milteny Biotec). Isolation of the lymphocytes of the lamina propria was performed by using the Lamina Propria Dissociation kit (mouse) from Milteny Biotec via gentleMACS^™^ dissociator following manufacturer’s instructions. Briefly, enzymatic digestion and solution is performed by combining different enzymatic and mechanical dissociation steps. Immediately, lymphocytes were isolated by MACS^®^ MicroBead separation. Blood lymphocytes were isolated by differential centrifugation and treatment with hypotonic eBioscience^™^ 1× RBC (red blood cell) lysis buffer (Thermo Fisher). Isolated lymphocytes were resuspended in TexMACS^™^ medium (Milteny Biotec).

Restimulation of all cells for cytokine analysis was performed by adding a mix of phorbol myristate acetate (PMA) and ionomycine as well as Brefeldin A (Golgi-Plug^™^) to block intracellular transportation processes for analysis. The following antibodies were used in a volume of 50 μL in the referred dilutions (if not stated otherwise, antibodies were manufactured by BioLegend): mLy-6G FITC (1:300, clone: 1A8), mCD64-PE (1:200, clone: X54-5/7.1), mLy-6C PC5.5 (1:300, clone: HK1.4, eBioscience), mCX3CR1 PECy7 (1:200, clone SA011F11), F4/80 APC (1:100, BM8), mCD11c AF700 (1:200, N418), m/hCD11b BV510 (1:300, M1/70), mCD45 APC/Cy7 (1:300, clone: 30-F11), m/hCD11b BV510 (1:300, clone M1/70), mCD3 APC-Cy7 (1:200, 17A2), mCD25 BV510 (1:200, PC61), mCD4 BV605 (1:200, clone: GK1.5), mCD8a BV650 (1:200, 53-6.7). Intracellular stainings were performed using the Cytofix/Cytoperm Plus Kit^™^ (BD) following the manufacturer’s protocol. The following antibodies were used: mIL-17A BV421 (1:100, clone: TC11-18H10.1), mIFN-y APC (1:100, XMG1.2), mIL-6 PE (1:100, MP5-20F3), mTNF-α AF700 (1:100, MP6-XT22), mFoxP3 AF700 (1:200, FJK-16s, eBioscience^™^), mGM-CSF APC-Fire750 (1:100, clone MP1-22E9). The measurements were performed on a CytoFLEX^™^ flow cytometer (both Beckman Coulter Inc.) and data analysis was conducted with FlowJo v10.8.1 Software (Becton Dickinson GmbH). Gating strategies are depicted in Supplementary Figures S1–S3.

### 2.8 Isolation of human peripheral blood mononuclear cells from EDTA blood

Eighteen millilitres blood were obtained from six stroke patients and six healthy controls. Stroke patients had a median age of 62.5 (IQR_25–75_ = 53.75–86.25) years and a median NIHSS of 3 (IQR_25–75_ = 0.75–12.75). The median age of the healthy controls was 32.5 (IQR_25–75_ = 29.5–47.5) years. One patient suffered from infection, but as his cytokine levels did not differ significantly from the other patients as tested via robust outlier (ROUT) method based on the false discovery rate (18), we did not exclude this patient from the analysis. Half of the stroke patients received intravenous thrombolysis, two others received mechanical thrombectomy. peripheral blood mononuclear cells (PBMC) were isolated applying density gradient centrifugation. After measuring the cell count (cell counter CASY, OLS omni life science), cells were resuspended in a calculated volume of freezing medium to obtain a concentration of 1 × 10^7^ cells/mL. The samples were stored under liquid nitrogen until further processing.

### 2.9 Stimulation assay with CLA

After isolation of PBMCs, CD4^+^ T-lymphocytes were isolated using the CD4^+^ T Cell Isolation Kit^™^, human and the MidiMACS^™^ cell separation system (Miltenyi Biotec, today BD Bioscience) following the manufacturer’s recommendations. The cells were adjusted to a concentration of 1 × 10^6^ cells/mL TheraPEAK^™^ X-VIVO^™^-15 Serum-free Hematopoietic Cell Medium (Lonza). A 96 well U bottom plate was coated with 5 mg/mL anti-CD3 (1:395, clone: OKT3, BioLegend) and anti-CD28 (1:250, clone, CD28.2, BioLegend) for 1 h at 37°C. After washing the plates 3 times with PBS, 6 wells per donor were seeded with 200,000 CD4^+^ T cells (in a volume of 200 μL X-VIVO medium). Three wells were additionally incubated with 50 μL CLA-mix (in pure form dissolved in ethanol to avoid contamination of oils) containing 50 mM cis-9,trans-11 CLA-isomer and 50 mM trans-10,cis-12 CLA-isomer (Sigma-Aldrich) for 72 h at 37°C. After 72 h, the supernatant was collected and analyzed for IL-17A, IFN-γ, TNF-α and IL-6 via Luminex xMAP technology (Thermo Fisher Scientific) following manufacturer’s protocol.

### 2.10 Study approval

The study on human blood samples was approved by the ethical committee of the Medical Association Westphalia Lippe and the University of Münster (AZ 2018-193-f-S).

### 2.11 Statistics

Statistical analyses were performed using GraphPad Prism version 10 (GraphPad Software, La Jolla, CA, United States) and R version 4.1.0 (R Foundation for Statistical Computing, Vienna, Austria). Automatic outlier analysis was performed by ROUT analysis in Prism as outlined above. All tests were performed at an *α* = 0.05.

Statistical tests were selected based on the results of normality testing (Shapiro–Wilk test) and equality of variances testing (*F*-test): specifically, independent samples t-tests were used when data met assumptions of normality and equal variances, whereas Mann–Whitney *U* tests were employed in cases where these assumptions were not met. *p*-value <0.05 was considered significant.

For the functional outcome assessed by the adhesive tape test, a one-sided *t*-test was calculated to investigate whether the difference between day 1 and 84 in the left paw was greater for CLA than for Ctrl (the difference was normally distributed). For the functional outcome assessed by the foot fault test between the CLA-and the standard diet were compared by repeated measures two-way-ANOVA with *post hoc* Tukey’s correction. Weight course was compared by repeated measures two-way-ANOVA with *post hoc* Śídák’s correction. Effects of CLA versus standard diet on corpus callosum metrics were investigated separately for animals with infarct sizes smaller and larger than the median infarct size using Mann–Whitney *U* tests. Statistical analysis of the flow cytometry data was performed with R version 4.2.2. Different cell types were compared between the two groups (CLA and control) across multiple tissues (blood, brain, LNc, LNm, siLP, and colP). To account for multiple comparisons in the flow cytometry analysis, the Benjamini–Hochberg (BH) procedure was applied to adjust the *p*-values. Analysis human blood samples were performed with paired mixed ANOVA and *post hoc* Śídák’s multiple comparison test.

### 2.12 Sample size calculation

Group sizes were calculated using the Sample Size Calculator software (Department of Statistics, University of British Columbia, http://www.stat.ubc.ca/~rollin/stats/ssize/n2.html). Behavioural data and histological examinations from previously conducted experiments were considered. In preliminary work, a functional, neurological deficit in the foot fault test of 16 ± 4% missteps was determined. With an expected reduction of the deficit to 13% following training therapy (approx. 20% reduction) and assuming a significance level of *α* = 0.05, this results in a test strength (1 − β) of 0.8 (80%) with a number of animals of *n* = 28 per group examined.

For our primary endpoint, the functional neurological deficit assessed by the foot fault test and the adhesive tape test, we used a total number of 37 animals (CLA group with photothrombotic stroke, *n* = 19; control group with photothrombotic stroke, *n* = 18). The secondary endpoints consisted of structural changes (MRI analysis; CLA group with photothrombotic stroke, *n* = 6; control group with photothrombotic stroke, *n* = 6) and immunological alterations (CLA group with photothrombotic stroke, *n* = 8; control group with photothrombotic stroke, *n* = 8). For our exploratory analysis, we did not exhaust the total number of animals per group precalculated which represents a potential limitation of our study.

This study followed the ARRIVE guidelines. The graphics were created using CorelDRAW Home & Student X8.

## 3 Results

### 3.1 CLA does not influence functional recovery after experimental photothrombotic stroke

**Figure 1:**
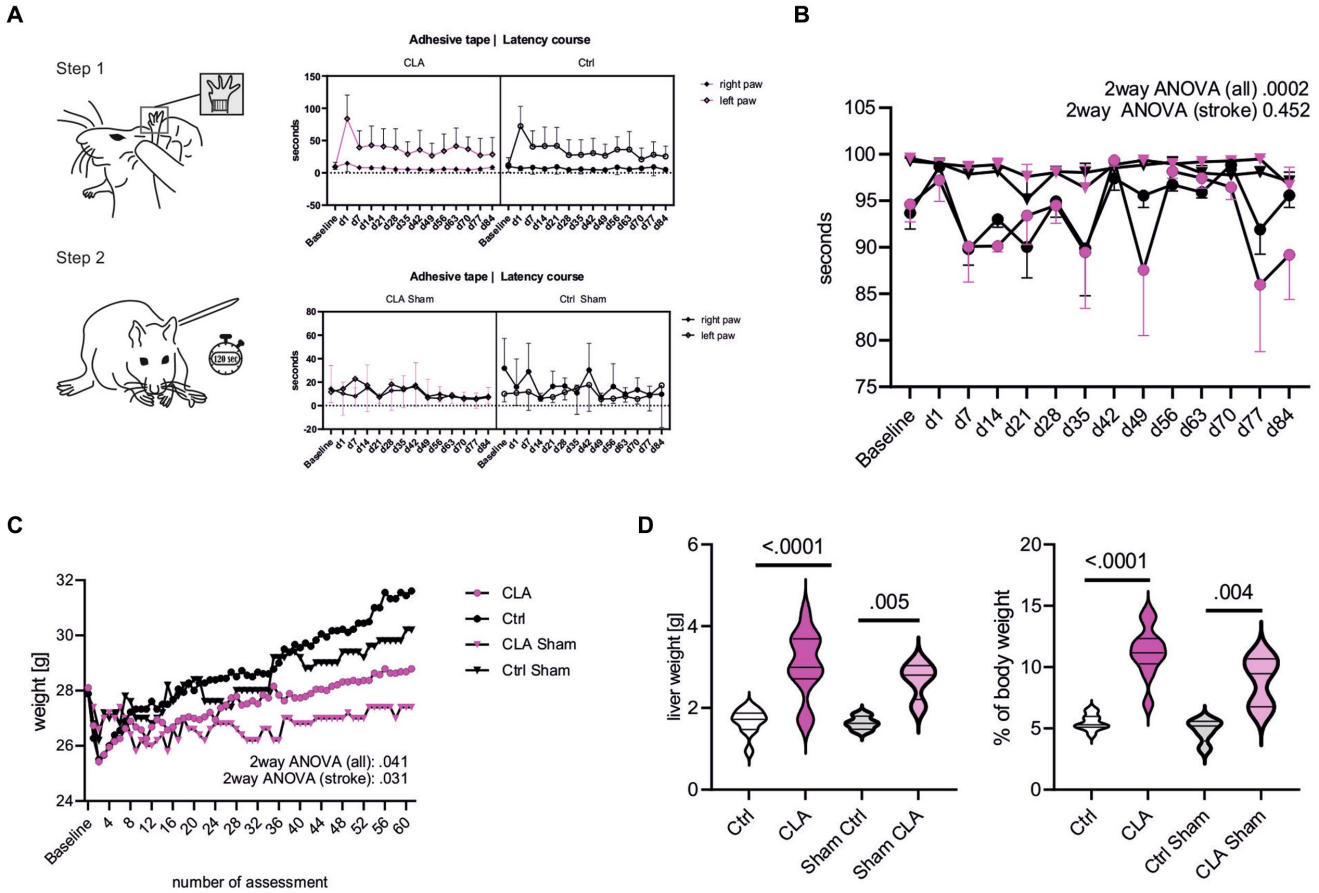
CLA supplementation during functional recovery after experimental photothrombotic stroke. Latency course for removal of the adhesive dots from the paretic paw (left paw) and healthy paw (right paw) over the experiment. A one-sided *t*-test was calculated to investigate whether the difference between day 1 and 84 in the left paw was greater for CLA than for Ctrl (the difference was normally distributed) **(A)**. Foot fault course comparing all groups including sham and only CLA stroke and control stroke. Values are depicted as mean with 95% confidence interval, courses were analyzed with two-way-ANOVA and *post hoc* Tukey’s correction (CLA *n* = 4, control *n* = 4, CLA sham *n* = 5, Ctrl sham *n* = 5) **(B)**. Weight course (**C**, assessment was performed 5 times per week after daily assessment during the first 2 weeks after operating procedure). Weight course was compared by repeated measures two-way-ANOVA with *post hoc* Śídák’s correction. Total liver weight in gram and liver weight in percent of total body weight, analyzed with independent samples *t*-test (**D**; Ctrl stroke *n* = 11, CLA stroke *n* = 12, Ctrl sham *n* = 4n CLA sham *n* = 4). Besides foot fault test and liver weight, following animal numbers: CLA *n* = 19, control *n* = 18; CLA sham *n* = 5, control sham *n* = 5. No outliers were excluded; unequal group numbers resulting from animals having died after induction of photothrombotic stroke. CLA, conjugated linoleic acid; Ctrl, control; d, day; g, gram.

To assess functional recovery, we performed the adhesive tape removal test reflecting sensorimotor deficits by asymmetric forelimb use, as detailed in the methods section. The course of removal of the adhesive dot from the paretic paw was not different from the course in the standard diet group after 90 days (Figure 1A). Additionally, we analyzed the difference between day 1 and day 84 in the left paw. The difference was non-significantly higher with an average effect size based on Cohen’s *d* in the CLA-group, indicating greater improvement (55.30 vs. 46.71 s; *p* = 0.266, *d* = 0.21). The foot fault course between the CLA and the control group subjected to photothrombotic stroke did not differ significantly (Figure 1B). The increasing course of weight of the animals in the group fed a CLA-rich diet was significantly lower than the one in the standard diet group over the 90 days (two-way ANOVA for animals subjected to photothrombotic stroke, *p* adj. = 0.031; Figure 1C). Livers were significantly enlarged in the CLA-group (1.82 vs. 3.54 g, *p* < 0.0001, Figure 1D left) and consequently accounted for a higher percentage of body weight (5.64 vs. 12.32%, *p* < 0.0001; Figure 1D right). We observed this in the sham animals as well, so liver weight was independently increased from photothrombotic stroke (1.63 vs. 2.68 g, *p* = 0.005, Figure 1D left) and accounted for a higher percentage of body weight as well (4.86 vs. 8.86%, *p* = 0.004, Figure 1D left) (Supplemental Table 1).

### 3.2 CLA improves the microstructural reorganization of white matter tracts

**Figure 2:**
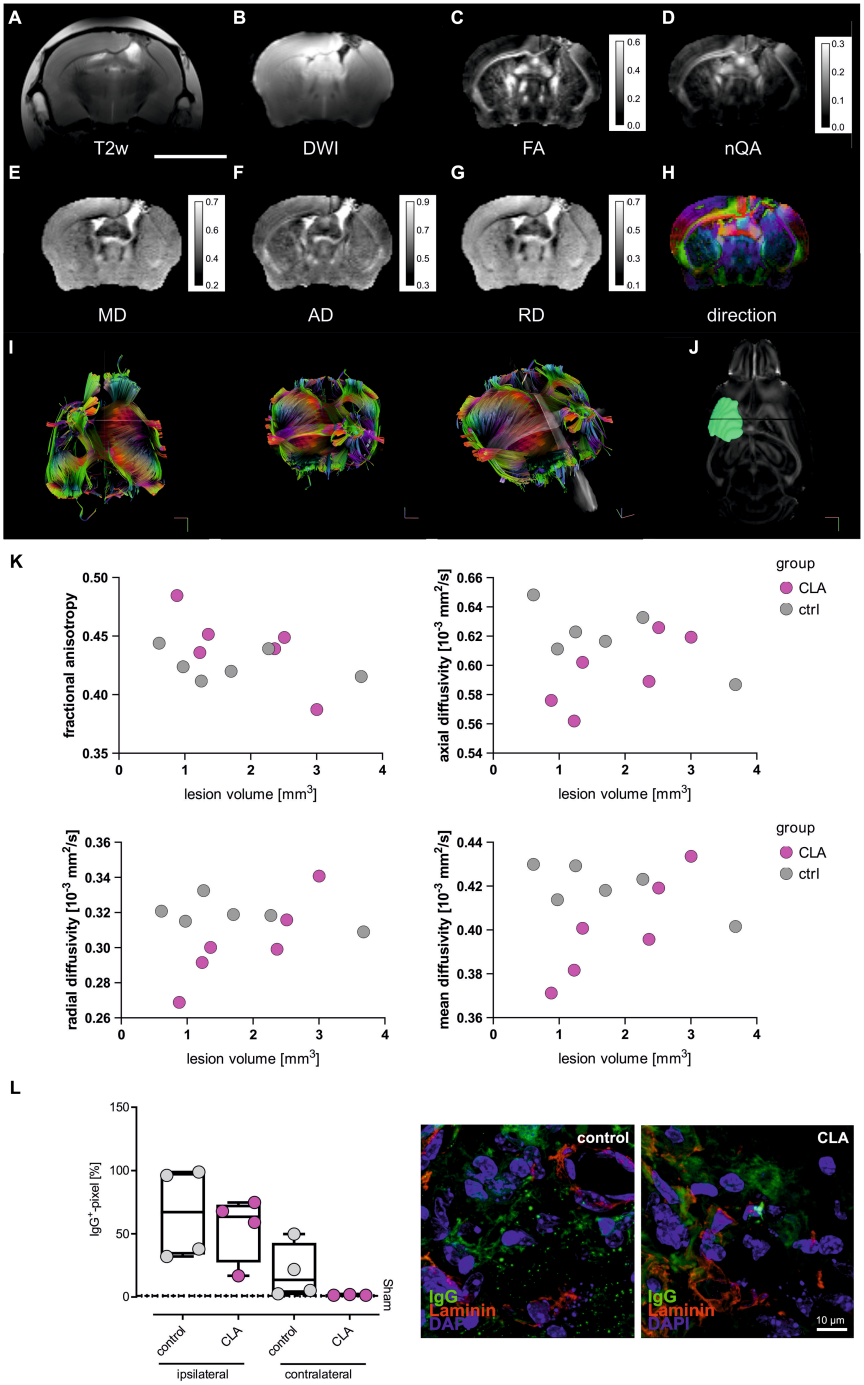
CLA affects diffusivity metrics in smaller infarcts. T2-weighted anatomical reference **(A)**. Diffusion weighted image as sum of all diffusion directions **(B)**. Mean diffusivity **(C)**. Fractional anisotropy **(D)**. Normalized quantitative anisotropy **(E)**. Axial diffusivity **(F)**. Radial diffusivity **(G)**. Color-coded principal diffusion direction (red: left-right; green: anterior-posterior; blue: dorsal-ventral) **(H)**. Scale bar = 5 mm. Diffusivities are given in ×10^−3^ mm^2^/s. Calibration bars are windowed to enhance tissue details (pixel values larger than maximum values on calibration bar are not resolved). Exemplary pictures of MRI tractography **(I)** and lesion mapping **(J)**. Man-Whitney-*U*-test of small (<1.53 mm^2^, *n* = 6) and large (>1.53 mm^2^, *n* = 6) infarcts of the structural metrics of the corpus callosum. It revealed a trend of higher fractional anisotropy and significant lower diffusivities for RD, AD and MD on small lesion volumes **(K)**. Box plots showing the mean for IgG^+^ cells per pixel for the ipsilateral and the contralateral side of the infarct in the corpus callosum region (left side). Values were analyzed with ordinary one-way ANOVA and *post hoc* Tukey’s correction (no significant differences). Exemplary immunohistochemical picture with IgG stained in green, Laminin as a marker for the vasculature in red and DAPI in blue (right side) (**L**, sham control and sham CLA *n* = 5 each, photothrombotic stroke control diet and CLA-diet *n* = 4 each). MRI, magnetic resonance imaging; T2w, T2-weighted; DWI, diffusion-weighted imaging; FA, fractional anisotropy; nQA, normalized quantitative anisotropy; MD, mean diffusivity; AD, axial diffusivity.

To investigate potential structural correlates of functional improvement after CLA supplementation, we performed deterministic fiber tracking via diffusion MRI measurements. We chose the corpus callosum (CC) as region of interest as it represents a brain region tightly packed with myelinated axons and thus is especially vulnerable to ischemia (11, 19, 20). The white matter repair is one critical element of long-term stroke recovery (21). The stroke lesion in the left somatosensory cortex shows deformed corpus callosum and the lateral ventricle in all images (Figures 2A-J). The diffusion sum (Figure 2B) is reduced in the area of the lesion, corresponding to low anisotropy values (Figures 2C,D) albeit at relatively high mean diffusivity (Figure 2E). High mean diffusivity is most likely caused by the inclusion of embedding agar or residual fluid, as this area also shows high axial (Figure 2F) and radial diffusivities (Figure 2G). The corpus callosum is characterized by low radial diffusivity and medium-to-high axial diffusivity, leading to prominent diffusion anisotropy. Consequently, direction-coded colour maps clearly highlight the corpus callosum as a subcortical structure with dominant left-right diffusion anisotropy (red in panel H; directions were derived from nQA, Figure 2H) ventral to the cingulum and other supra-cortical white matter that run anterior-posterior in each respective hemisphere (green) (22).

As we visually had the impression of differential effects of CLA depending on infarct size, we compared animals with small infarcts (<median = 1.53 mm^2^) and large infarcts (>median = 1.53 mm^2^). For small infarcts, we found significantly improved microstructural regeneration among animals that received a CLA-enriched diet. We observed a trend towards increased fractional anisotropy (FA) of the corpus callosum (0.43 vs. 0.46; *p* = 0.100, Figure 2K), indicating improved microstructural properties of the white matter tracts. In line with the trend for FA, axial diffusivity (AD) and radial diffusivity (RD) of the corpus callosum were significantly lower (AD: 0.63 vs. 0.58, *p* = 0.050; RD: 0.32 vs. 0.29, *p* = 0.050, Figure 2K), indicating enhanced myelination. Finally, the mean diffusivity (MD) of the corpus callosum was also significantly lower (0.42 vs. 0.38, *p* = 0.050, Figure 2K), indicating reduced vasogenic edema (15). We observed no significant microstructural changes for large infarcts (Figure 2K). Raw data is provided in the supplement (Supplemental Table 2).

Overall, MRI measurements thus indicate that a CLA-enriched diet enhances the microstructural reorganization of connecting white matter tracts after stroke in small infarcts.

As we interpreted the lower MD-values as reduced vasogenic edema, we additionally performed immunohistochemical experiments of IgG presence after 90 days in the corpus callosum in another cohort of animals. IgG should not be present in the brain given an intact selectivity of the blood-brain-barrier. As expected, no IgG was found in the sham animals, while it was still detected in the experimental animals after 90 days. However, the visual difference between control animals and those fed with CLA was not significant (Figure 2L, the depicted stained infarcts would theoretically be categorized into small infarcts).

### 3.3 CLA-enriched diet does not affect immune cell differentiation along different compartments

As we observed an improved microstructural reorganization following CLA-rich diet, we next aimed to assess immunological correlates. First, we analyzed immune cell differentiation of myeloid cells and T cells along the different compartments of the gut-brain-axis, including the lamina propria of the small intestine and colon as well as mesenteric lymph nodes (gut), the peripheral blood and the draining cervical lymph nodes.

**Figure 3:**
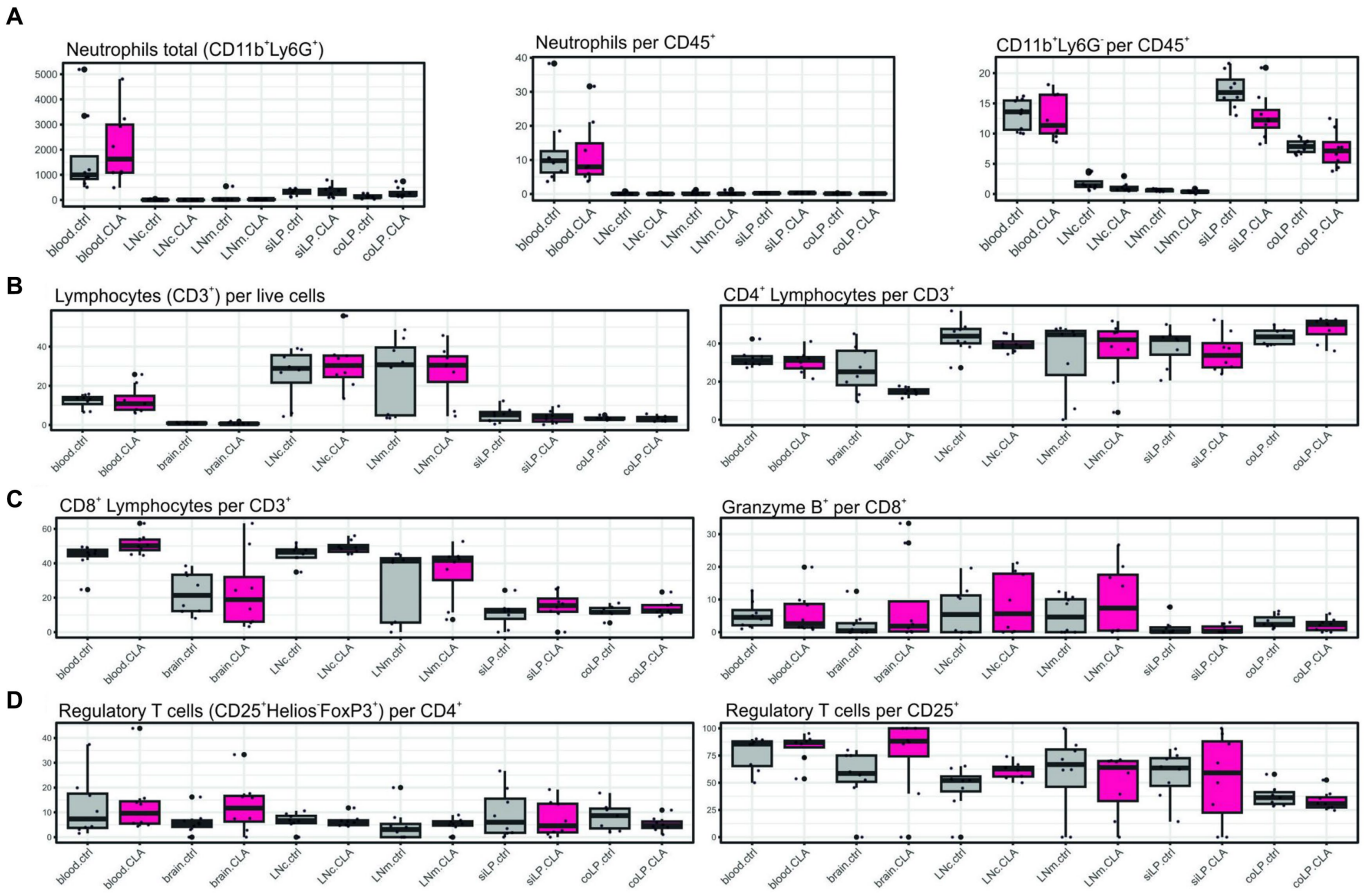
CLA without affection on differentiation of different immune cells subsets. Immune cells of different organs were stained with surface markers for determination of their differentiation via flow cytometry. Total CD11b^+^ Ly6G^+^ cells indicating neutrophils, neutrophils per CD45^+^ cells and CD11b^+^ Ly6G-per CD45^+^ cells indicating monocytes/macrophages, eosinophils and dendritic cells **(A)**. CD3^+^ cells indicating lymphocytes per live cells and CD4^+^ lymphocytes per CD3^+^ **(B)**. CD8^+^ lymphocytes per CD3^+^ and Granzyme B^+^ cells per CD8^+^ cells **(C)**. CD4^+^ CD25^+^ Helios-FoxP3^+^ cells indicating regulatory T cells per CD4^+^ and per CD25^+^ cells **(D)**. *n* per group = 8. Data are presented as box plots. Samples were collected at 90 days post stroke. Analysis performed with unpaired *t*-test or Mann–Whitney test respecting normality and *post hoc* Benjamin–Hochberg correction. LNc, cervical lymph nodes; LNm, mesenterial lymph nodes; siLP, lamina propria of the small intestine; coLP, lamina propria of the colon.

We did not obtain significant alterations (Figures 3A-D). Albeit, total neutrophil count tended to increase in the blood upon CLA-treatment (1625.46 vs. 998.46; *p* adj. = 0.852, Figure 3A, left) whereas the CD11b^+^ Ly6G-populations consisting of monocytes/macrophages, dendritic cells and eosinophils visually seemed to decrease in the blood (13.60 vs. 11.35; *p* adj. = 0.935, Figure 3A, right). Regulatory T cells visually appeared to increase in the brain, without significancy (11.72 vs. 5.51 per CD4^+^; *p* adj. = 0.936; 88.20 vs. 58.55 per CD25^+^; *p* adj. = 0.936) (Figure 3D).

### 3.4 Cytokine levels are not affected either in myeloid nor in T cells upon CLA-enriched diet

**Figure 4:**
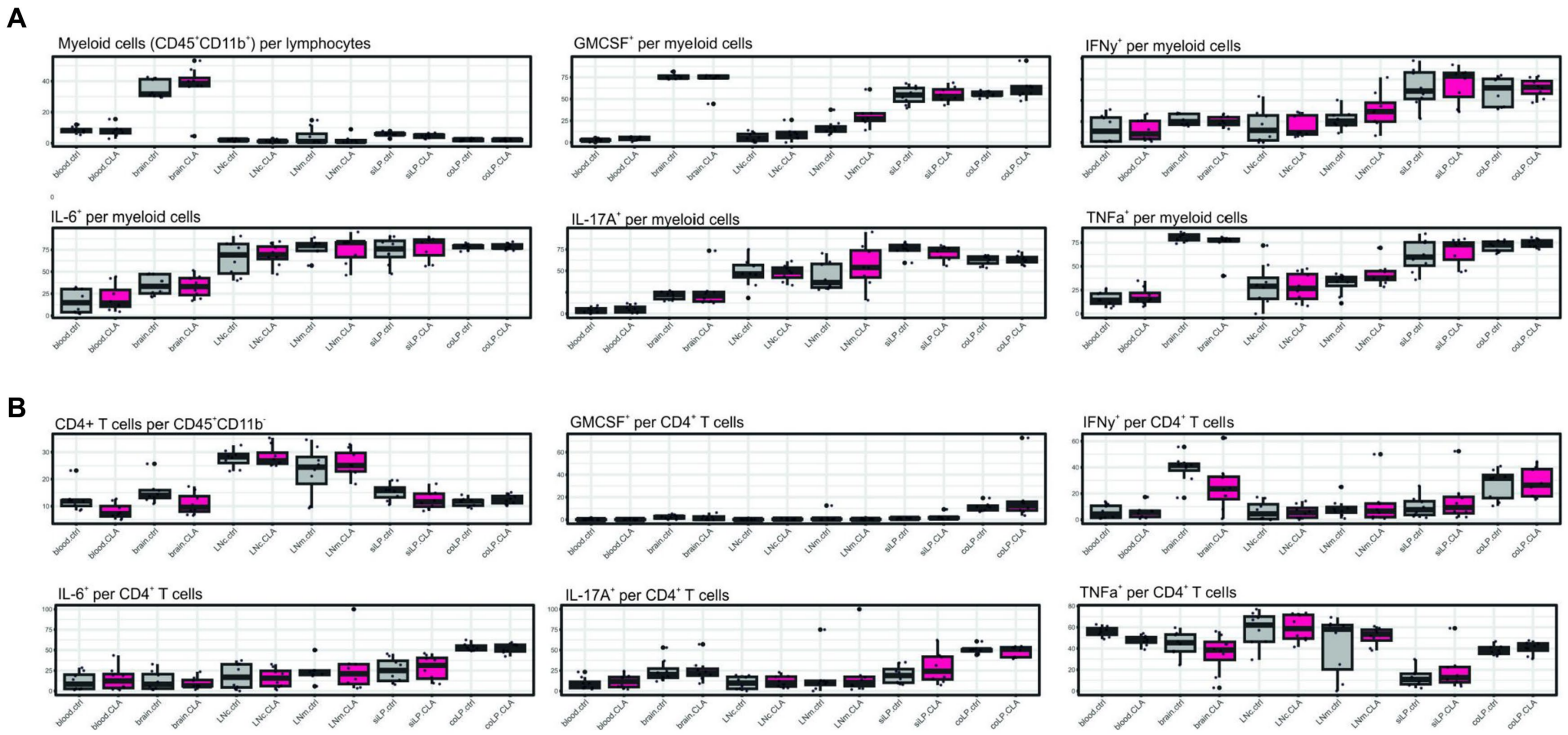
CLA does not affect cytokine positivity in myeloid or CD4^+^ T cells. Cytokine analysis after restimulation of isolated cells with PMA and ionomycin and treatment with brefeldin A. CD45^+^ CD11b^+^ cells indicating myeloid cells per lymphocytes and respective cytokine positivity per myeloid cells **(A)**. CD45^+^ CD11b-CD4^+^ cells indicating CD4^+^ T cells per CD45^+^ CD11b-and respective cytokine positivity per CD4^+^ T cells. *n* per group = 8. Data are presented as box plots. Samples were collected at 90 days post stroke. Analysis performed with unpaired *t*-test or Mann–Whitney test respecting normality and *post hoc* Benjamin–Hochberg correction. GM-CSF, granulocyte macrophage-colony stimulating factor; IFN-γ, interferon γ; IL-6, interleukin 6; IL-17A, interleukin 17A; TNF-α, tumor necrosis factor α.

Furthermore, we investigate possible cytokine alterations in myeloid and T cells upon CLA diet. We were not able to find significant alterations potentially correlating with improved microstructural reorganization on this level (Figures 4A, B).

### 3.5 CLA suppresses pro-inflammatory cytokine secretion of human lymphocytes *in vitro*

To assess a potential translational relevance of CLA-mediated effects for stroke patients, we next investigated the *in vitro* effect of CLA on human lymphocytes. We thus isolated CD4^+^ T lymphocytes from peripheral mononuclear cells of healthy donors and stroke patients.

**Figure 5:**
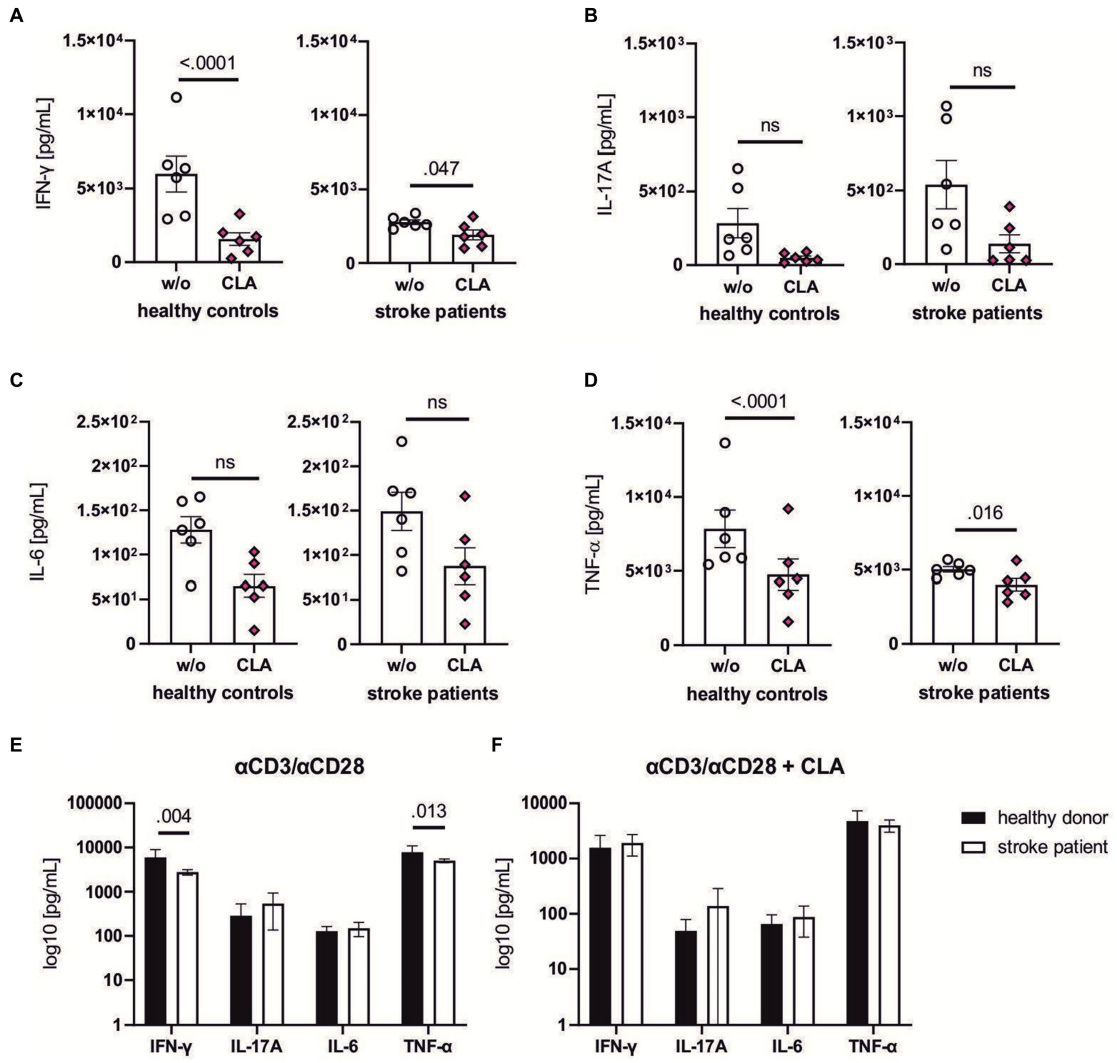
CLA dampens pro-inflammatory cytokine levels of stroke patients and healthy donors *in vitro*. CD4^+^ T lymphocytes from healthy controls and stroke patients (day 1–4 after ischemic stroke) were isolated, stimulated with αCD3/αCD28 antibodies and incubated for 72 h with or without 50 mM 9cis,11trans-and 50 mM 10trans,12cis-CLA. Supernatant was collected and the levels of the following cytokines were assessed via Luminex xMAP technology: IFN-γ **(A)**, IL-17A **(B)**, IL-6 **(C)** and TNF-α **(D)**. 200,000 cells per seeded well, *n* = 6 each with seeding into three wells per w/o and stimulation with CLA. The baseline levels between healthy controls were significantly higher in the healthy control group after αCD3/αCD28 stimulation for IFN-γ and TNF-α **(E)**, indicating effects of stroke-induced immunosuppression in the patient cohort. The cytokine levels after CLA stimulation did not differ significantly **(F)**. *n* = 6. Data shown as box plots **(A–D)** and mean ± SEM **(E,F)**. Analysis performed with paired mixed ANOVA and *post hoc* Śídák’s multiple comparison test. ROUT analysis revealed no outliers. IFN-γ, interferon γ; IL-17A, interleukin 17A; IL-6, interleukin 6; TNF-α, tumor necrosis factor α.

As the stroke represents a strong inflammatory event, we performed anti-CD3/anti-CD28 (αCD3/αCD28) treatment of all samples to imitate the inflammatory stimulus. Half of the cells were concomitantly treated with CLA (Figures 5A-F).

Most importantly, the lymphocytes of stroke patients and healthy donors were responsive to the CLA treatment: Lymphocytes of stroke patients showed a significantly reduced production of pro-inflammatory cytokines IFN-γ (2775.0 vs. 1915.0 pg/mL, *p* adj. =0.047, Figure 5A), and TNF-α (5018.0 vs. 4005.0 pg/mL, *p* adj. = 0.016, Figure 5D). Similarly, the concentrations of IFN-γ (5989.0 vs. 1571.0 pg/mL, *p* adj. <0.0001, Figure 5A) and TNF-α (7841.0 vs. 4761.0 pg/mL, *p* adj. <0.0001, Figure 5D) of healthy controls were decreased after incubation with CLA for 72 h.

Comparing the secretion of pro-inflammatory cytokines between healthy donors and stroke patients upon stimulation with αCD3/αCD28, stroke patients were less responsive as indicated by reduced secretion of IFN-γ (5989.0 vs. 2775.0 pg/mL, *p* adj. = 0.004, Figure 5E) and TFN-α (7841.0 vs. 5018.0 pg/mL, *p* adj. = 0.013, Figure 5E). Upon combined αCD3/αCD28-and CLA-treatment, the cytokine levels responded similarly.

Altogether, these findings demonstrate CLA has the capacity to partially suppress pro-inflammatory cytokine production in human lymphocytes.

## 4 Discussion

Our results suggest that CLA supplementation enhances the microstructural reorganization of interhemispheric white matter tracts in small infarcts, even though it did not alter functional outcomes in our experiments. In particular, CLA-supplementation had significant effects on structural metrics of the corpus callosum in small-sized infarcts indicating a more organised tissue reflected by pronounced directional diffusivity. We analyzed the corpus callosum as Wallerian degeneration occurs in the weeks after ischemic stroke as a correlate of distal degeneration of the axon and its myelin sheath and correlates with functional impairment. It can be assessed by determining anisotropy via diffusion tension imaging (23). Immune homeostasis is relevant for remyelination capacities by oligodendrocytes (24) which is why changes in white matter are of interest after an ischemic lesion in the gray matter as well. As a limitation of this exploratory study, we were only able to analyze post-mortem MRI data.

Immunological assessments revealed no significant alterations after 90 days. Our hypothesis proposed that changes in the gut microbiome could positively influence immune cell composition and potentially regulate immune responses through the gut-brain axis. The gut microbiome and its diversity represent not only a risk factor for experimental and clinical stroke but is also relevant for stroke outcome. Most likely, immune cells contributing to secondary neurodegeneration (such as IL-17 secreting γδ T cells) as well as immune cells exerting a neuroprotective and regeneration-enhancing role (such as IL-10 secreting T_regs_) are primed in the intestine and find their way into the central nervous system (10, 25). Age, antibiotics and ischemic stroke are some factors associated with reduced gut microbiome diversity (10) which is why the effect of nutritional elements having the potential of skewing the immune reaction into a protective direction are of interest in research on stroke recovery.

However, *in vitro* analyses of human blood samples of healthy controls and stroke patients incubated with CLA showed a significantly decreased production of IFN-γ and TNF-α, and a visual decrease of IL-17A and IL-6. IL-17 is an important cytokine for neutrophil and monocyte recruitment and amplification of the immune response. Inhibition of the early response of dendritic cells significantly ameliorated experimental stroke outcome measured by a neurological scoring after Bederson (3, 26). IL-6 is associated with recurrent stroke risk and poorer functional outcomes (as reflected by the mRS score within 1 year) (27). However, the relevance of IL-6 is not restricted to post-stroke pathophysiology: a recent meta-analysis showed a linear positive relationship between IL-6 serum levels and the risk of suffering from ischemic stroke (28). IL-6 is a downstream cytokine of IL-1β. Interestingly, blocking the latter during the CANTOS trial in cardiovascular disease did result in a particularly positive effect when analysed for the lowering of plasma IL-6 levels (29). The effect of IL-6 is relevant due to its contribution to atherosclerosis and vulnerable vascular lesions (30). The link between IFN-γ and atherosclerosis formation is well established. IFN-γ affects macrophages promoting lipid uptake and consecutive formation of foam cells and it contributes to the upregulation of endothelial adhesion molecules (31). Furthermore, it also maintains the endothelial inflammatory response by recruitment of macrophages and T cells via chemokines including CC-chemokine ligand 2 (CCL-2), monocyte chemotactic protein-1 (MCP-1) and CXCL16 (32).

An unexpected observation were the comparably lower basal levels of TNF-α and IFN-γ in stroke patients compared to controls. In several preclinical studies the acute increase of cytokines in the hyperacute (<4 h) phase has been shown (33, 34). A study on human serum levels found that whereas TNFR1 and TNFR2 levels increased in the acute phase (<8 h), TNF levels in the plasma did not change at 8 and 72 h (35). IFN-γ is known to be elevated in the acute phase of stroke with a peak at 6–24 h and nearly normalisation at 48–96 h (36). A potential explanation for our observation could be that we obtained our blood samples after the acute elevation of cytokines (between day 2 and 5), during the phase of stroke-induced immunosuppression. This phase is characterized by an increase in IL-10 and IL-4 levels, which consequently leads to a reduction in pro-inflammatory cytokines (37, 38). Thus, it is possible that the determination of TNF-α and IFN-γ in our experimental design occurred too late, and we may have missed the early peak of these cytokines.

So far, CLA has not been tested in experimental stroke models. In various other autoimmune diseases, it has shown to skew the immune response into a rather regulatory phenotype. In different animal models of atherosclerosis, the dietary supplementation of CLA has not only led to halting of plaque progression but was also capable of inducing regression of formed plaques. This was accompanied by a reduction of plasma levels of cholesterol, LDL and triglycerides (6). Inhibited monocyte migration to MCP-1 mediated by PPAR-γ, a general upregulation of PPARs within the vasculature and a downregulation of pro-inflammatory genes were shown upon CLA-feeding (6, 39). In experimental spontaneous hypertensive disorder, CLA was capable of significantly reducing blood pressure values after a feeding period of 4 weeks, which was accompanied by a rise in adiponectin (40). This hormone exhibits antidiabetic and antiatherogenic properties, can be induced by CLA and can reduce obesity-related hypertension (41). We observed a significant decrease of body weight accompanied by increased liver size and weight in the CLA groups (sham and photothrombotic stroke). This changing effect on body composition and weight loss upon dietary CLA enrichment has been extensively studied and attributed to enhanced fatty acid oxidation, increased lipolysis as well as enhanced activity of brown adipose tissue with promoted expression of uncoupling protein 1 (UCP1) due to the trans-10,cis-12 (t10,c12) isoform (42, 43). Furthermore, we observed an enlarged liver size in the CLA treated group. This effect of hyperplasia without a change of liver composition has been investigated by Miranda et al. (43) who attributed this hepatomegaly to the trans-10,cis-12 (t10,c12) isoform as well. This effect was not associated with steatosis.

We neither observed strong effects on immune cell nor on cytokine composition in our animal model. Possibly, this effect is attenuated by the long-term approach of the study, as after a certain phase an equilibrium between the immunological response to stroke and the effects of CLA is established and here, we only observed the end points. This hypothesis might be supported by the reducing effect on pro-inflammatory cytokines we observed after CLA stimulation of T cells from healthy donors and stroke patients. The attenuated cytokine response upon αCD/αCD28-stimulation in stroke patients compared to healthy donors could be explained by stroke-induced immunosuppression, which occurs particularly in the subacute phase (3 to 7 days after the ischemic event) (44, 45). One limitation of our exploratory study is that there is a risk of false-neutral results due to the small sample size. Another limitation is that although the daily availability of the CLA-supplemented feed was controlled, the exact amount of CLA consumed will have varied between animals.

In summary, we here studied the potential efficacy of a regeneration-promoting therapy that is considered well-tolerated (8) and accessible to all stroke patients, regardless of dysphagia or severity of the condition. To date, physiotherapy outside the acute phase is the only evidence-based therapy to improve functional levels (44, 46). However, this therapy relies on the intrinsic motivation of the patient and its success is strongly influenced by psychological factors such as depression (47). Furthermore, current research is addressing potential cerebroprotective substances after reperfusion therapies as restoration of blood flow bears the risk of delivering inflammatory immune cells into the vulnerable region. This effect is very likely responsible for secondary deterioration or long-term sequelae after stroke despite successful recanalization (48). Dietary interventions with potential immunomodulatory effects represent an interesting additional approach to recanalization therapies and require further investigation.

As a main finding, our study shows that CLA enhances microstructural reorganization of the white matter tracts in small infarcts and attenuates the inflammatory response of human T cells *in vitro*.

## Data Availability

The raw data supporting the conclusion of this article (excluding the supplementary material) will be made available by the authors, without undue reservation.

## Footnotes

^1^ http://dsi-studio.labsolver.org/

## Supplementary material

Supplementary Figure S1 Gating strategy for myeloid cells. Immune cells in the respective organs (blood, LNc, LNm, siLP, coLP) were investigated by flow cytometry and identified as CD45 expressing cells. CD45^+^ subsets were separated into CD11b^+^ Ly6G^+^ (neutrophils) and CD11b^+^ Ly6G-cells (monocytes/macrophages, dendritic cells II, eosinophils). CD11b-cells were further subseparated into CD11c^+^ cells (dendritic cells I). LNc, cervical lymph nodes; LNm, mesenteric lymph nodes; siLP, lamina propria of the small intestine; coLP, lamina propria of the colon.

Supplementary Figure S2 Gating strategy for regulatory T cells. Live cells were identified by Zombie and CD3^+^ lymphocytes were gated. These were further separated into CD8^+^ and CD4^+^ lymphocytes. Within CD8^+^ lymphocytes, Gzmb was identified. Within CD4^+^ lymphocytes, CD25^+^ and FoxP3^+^ cells were gated. LNc, cervical lymph nodes; LNm, mesenteric lymph nodes; siLP, lamina propria of the small intestine; coLP, lamina propria of the colon.

Supplementary Figure S3 Gating strategy for cytokines. Immune cells were identified by CD45 and separated by CD11b^+^ (myeloid cells) and CD11b-and consecutive CD4^+^ cells (T cells). For both cell populations, IL-6, IL17-A, GM-CSF, TNF-α and IFN-γ were stained in the respective population and organ. LNc, cervical lymph nodes; LNm, mesenteric lymph nodes; siLP, lamina propria of the small intestine; coLP, lamina propria of the colon; IL-6, interleukin-6; IL17A, interleukin-17A; GM-CSF, granulocyte macrophage-colony stimulating factor; TNF-α, tumor necrosis factor-alpha; IFNγ, interferon-gamma.

SUPPLEMENTARY TABLE S1 Raw data of the Adhesive Tape test, the Neuroscore, the Foot Fault Test and Weight course over the experiment.

SUPPLEMENTARY TABLE S2 Raw data of MRI experiments.
