# Supplementary figures and images for: A dietary intervention with conjugated linoleic acid enhances microstructural white matter reorganization in experimental stroke
Source: Front Neurol. 2024 Sep 20;15:1341958. doi: 10.3389/fneur.2024.1341958 (PMC11449868; doi:10.3389/fneur.2024.1341958)

# Supplement Figure 1

## A blood

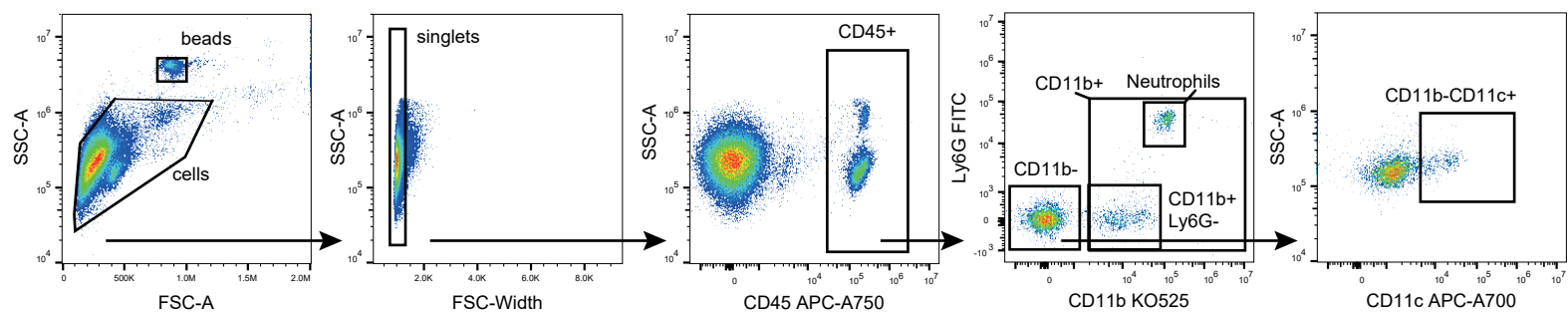

## B LNc

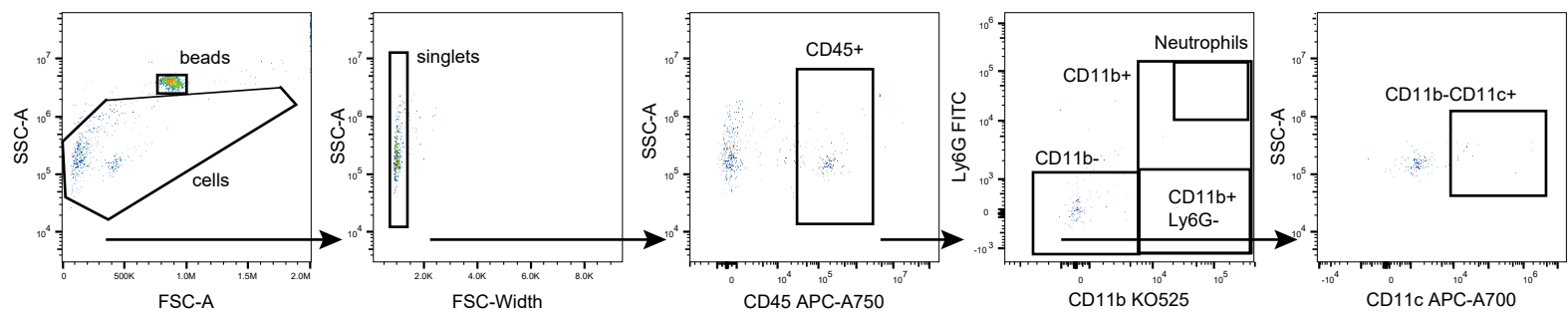

## C LNm

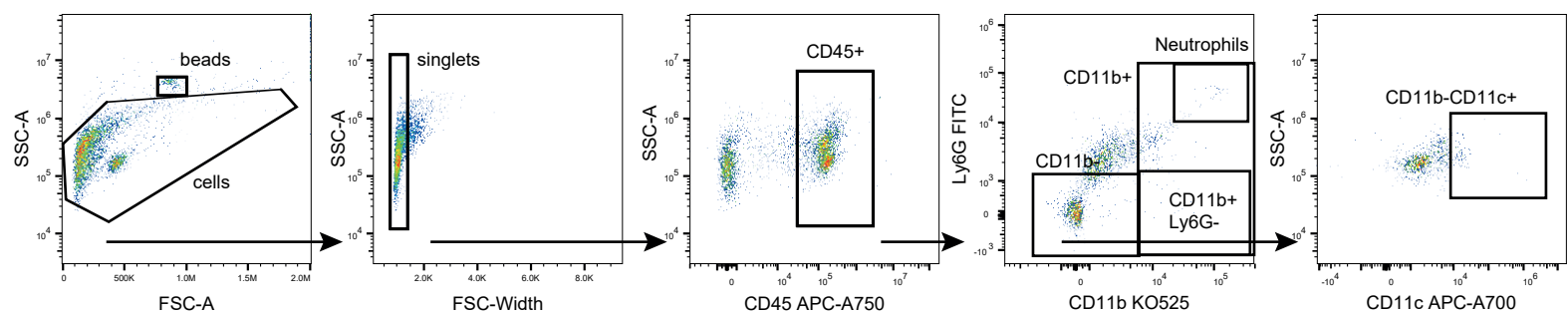

## D siLP

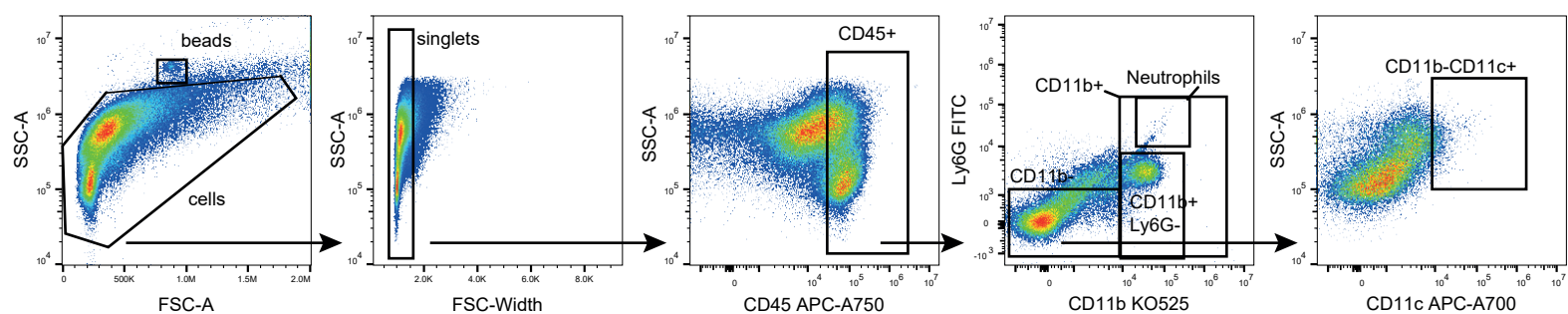

## E coLP

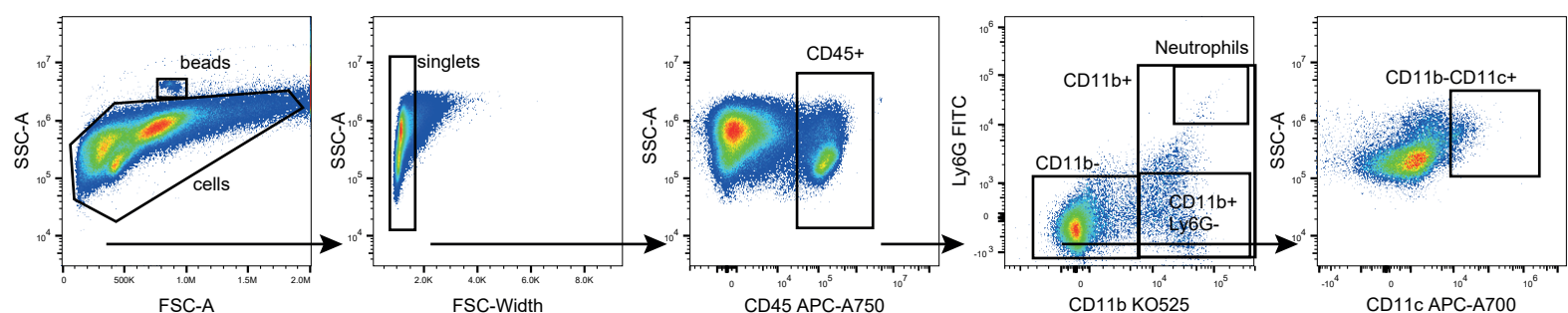

Supplement: Supplementary Figure S1 — Gating strategy for myeloid cells. Immune cells in the respective organs (blood, LNc, LNm, siLP, coLP) were investigated by flow cytometry and identified as CD45 expressing cells. CD45+ subsets were separated into CD11b+ Ly6G+ (neutrophils) and CD11b+ Ly6G-cells (monocytes/macrophages, dendritic cells II, eosinophils). CD11b-cells were further subseparated into CD11c+ cells (dendritic cells I). LNc, cervical lymph nodes; LNm, mesenteric lymph nodes; siLP, lamina propria of the small intestine; coLP, lamina propria of the colon. [file Data_Sheet_2.PDF]

Supplement Figure 2

A blood

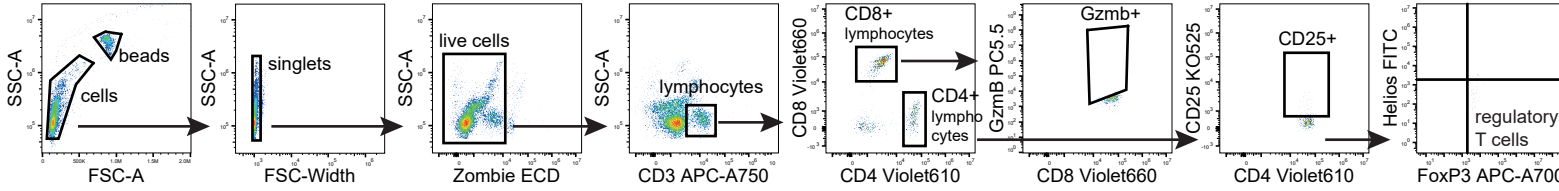

B brain

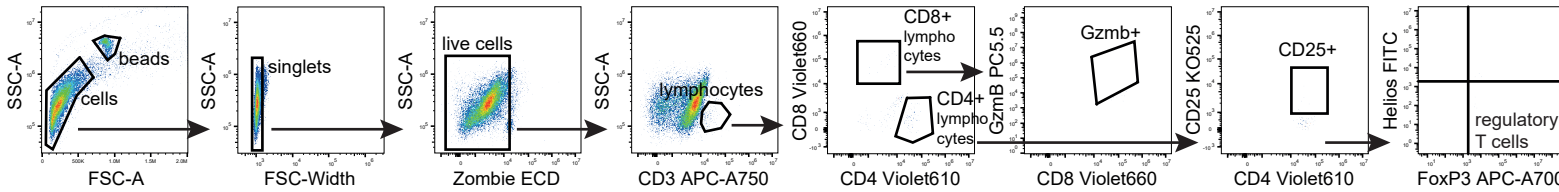

C LNc

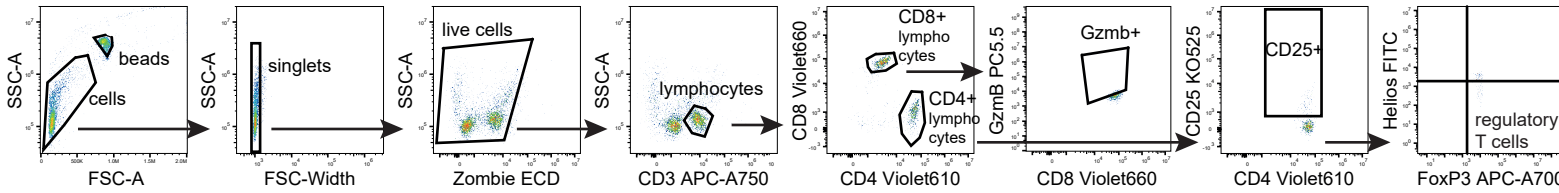

D LNm

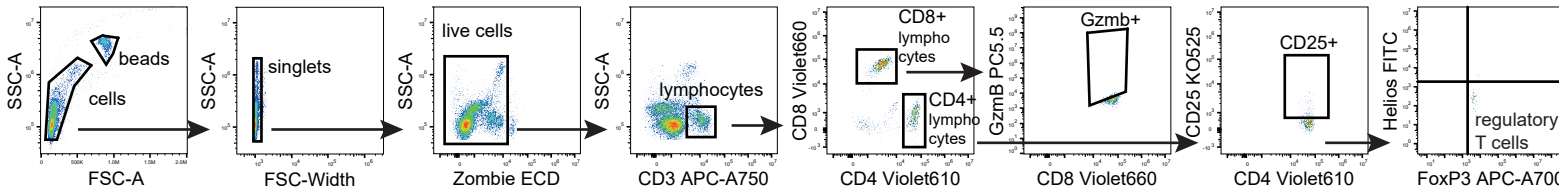

E siLP

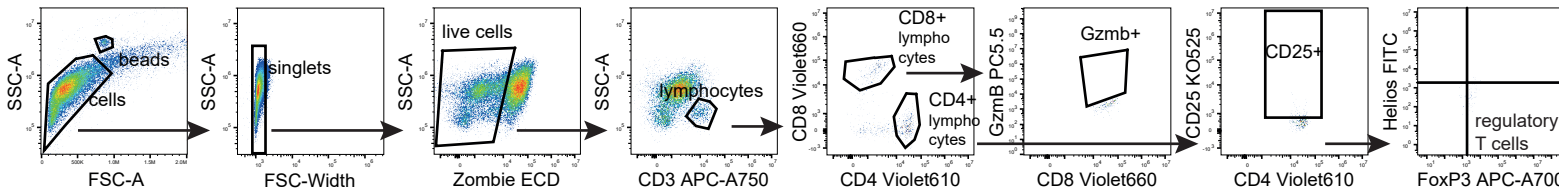

F coLP

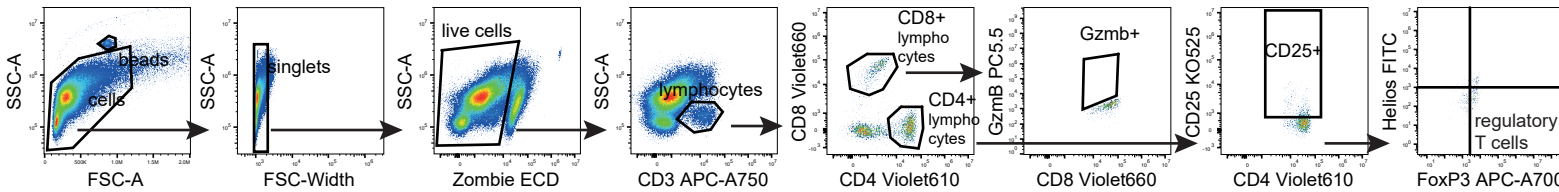

Supplement: Supplementary Figure S2 — Gating strategy for regulatory T cells. Live cells were identified by Zombie and CD3+ lymphocytes were gated. These were further separated into CD8+ and CD4+ lymphocytes. Within CD8+ lymphocytes, Gzmb was identified. Within CD4+ lymphocytes, CD25+ and FoxP3+ cells were gated. LNc, cervical lymph nodes; LNm, mesenteric lymph nodes; siLP, lamina propria of the small intestine; coLP, lamina propria of the colon. [file Data_Sheet_3.PDF]

Supplement Figure 3

## A blood

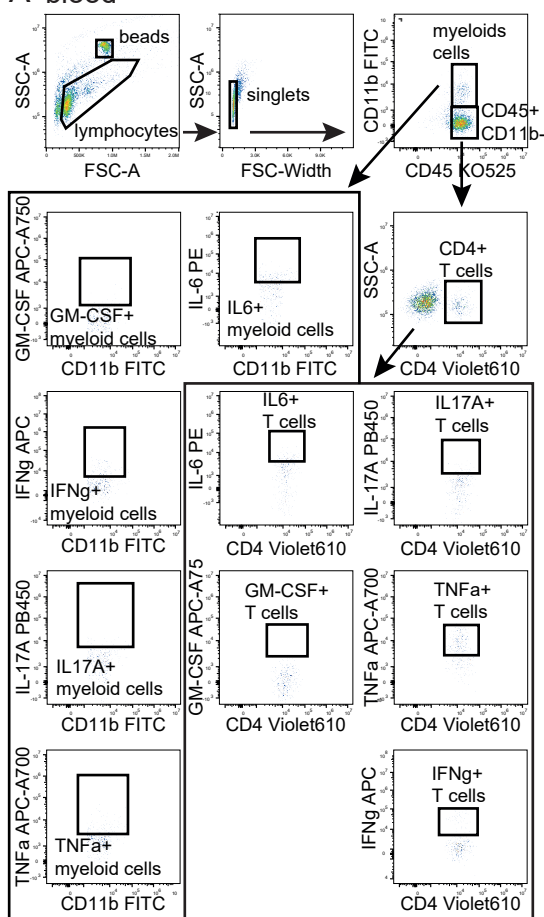

## B brain

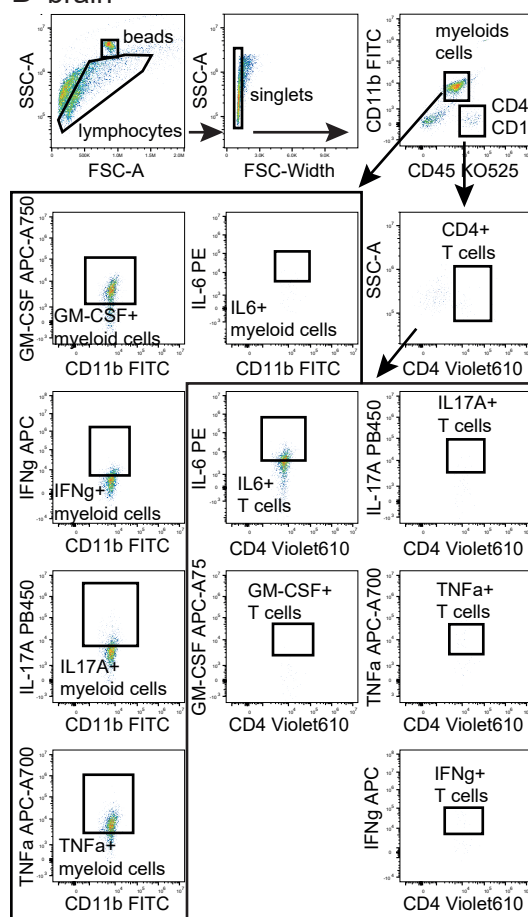

## C LNC

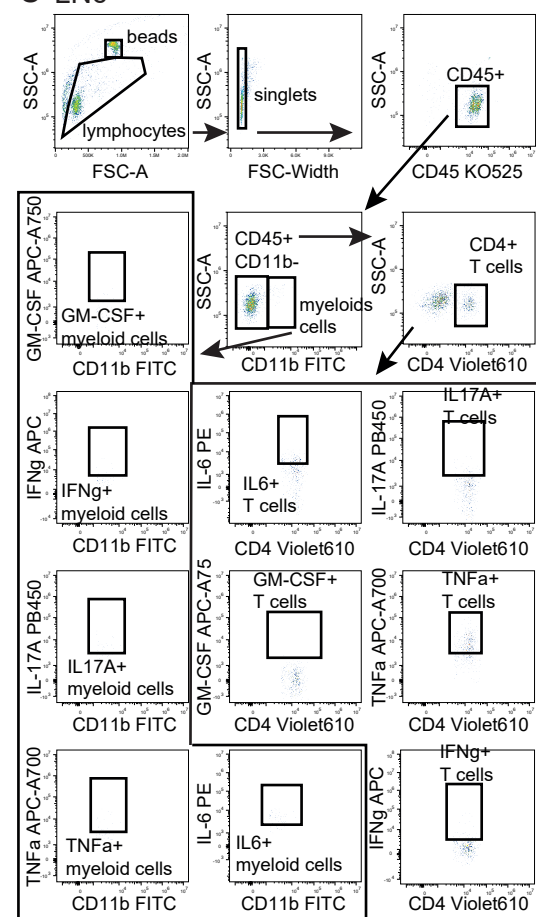

## D LNm

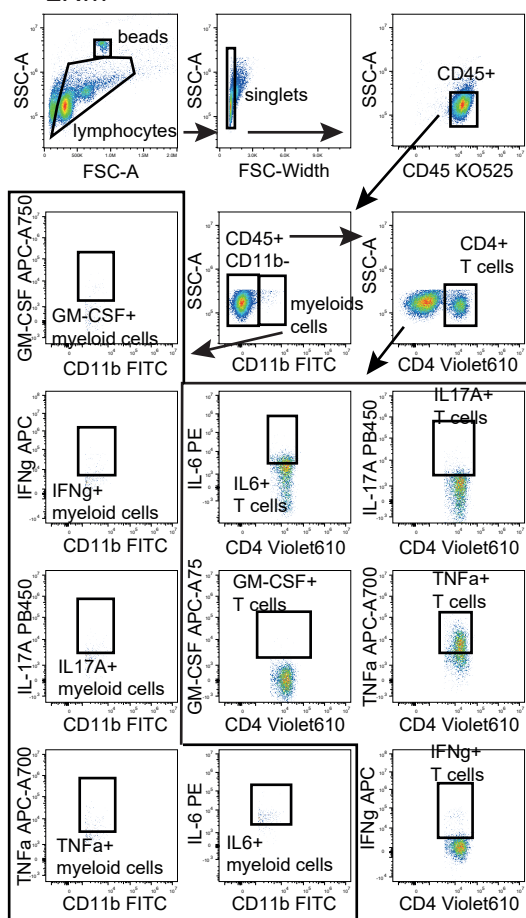

## E siLP

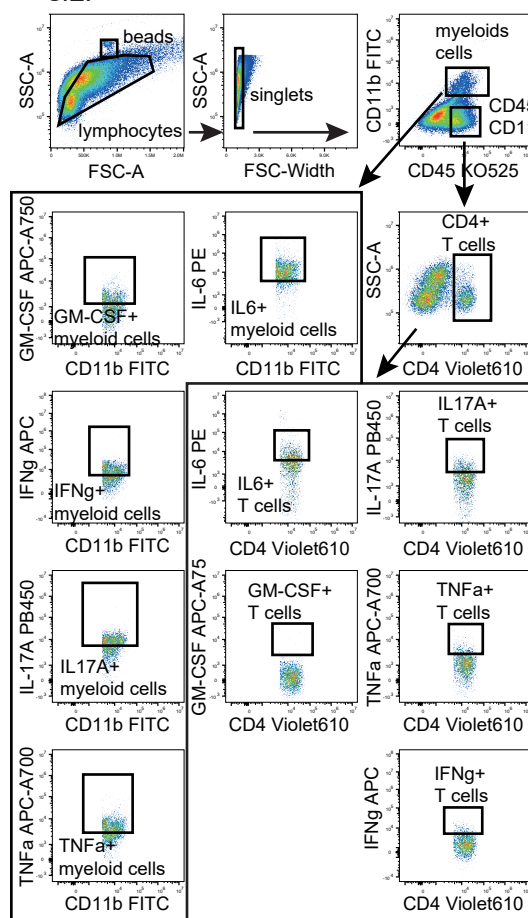

## F coLP

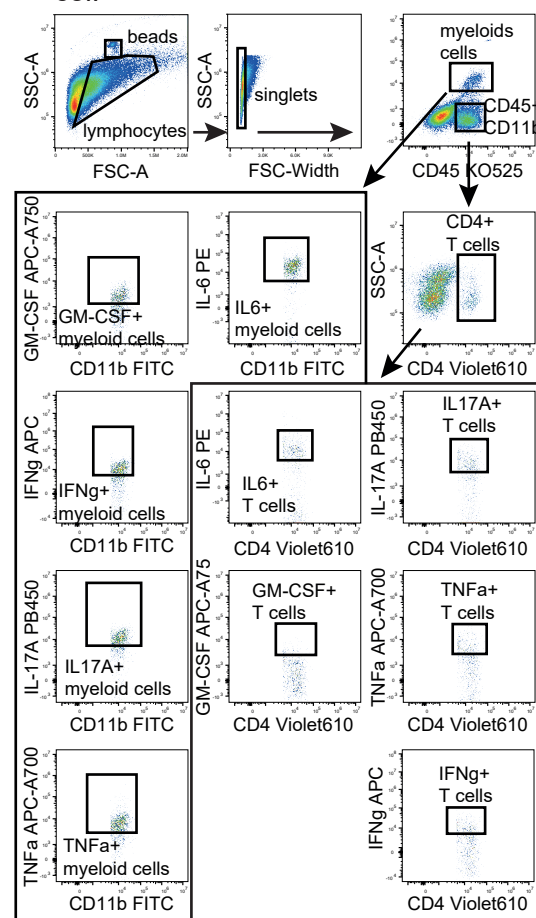

Supplement: Supplementary Figure S3 — Gating strategy for cytokines. Immune cells were identified by CD45 and separated by CD11b+ (myeloid cells) and CD11b-and consecutive CD4+ cells (T cells). For both cell populations, IL-6, IL17-A, GM-CSF, TNF-α and IFN-γ were stained in the respective population and organ. LNc, cervical lymph nodes; LNm, mesenteric lymph nodes; siLP, lamina propria of the small intestine; coLP, lamina propria of the colon; IL-6, interleukin-6; IL17A, interleukin-17A; GM-CSF, granulocyte macrophage-colony stimulating factor; TNF-α, tumor necrosis factor-alpha; IFNγ, interferon-gamma. [file Data_Sheet_4.PDF]
